# Human ATG4 autophagy proteases counteract attachment of ubiquitin-like LC3/GABARAP proteins to other cellular proteins

**DOI:** 10.1074/jbc.AC119.009977

**Published:** 2019-07-17

**Authors:** Alexander Agrotis, Lucas von Chamier, Harriet Oliver, Koshiro Kiso, Tanya Singh, Robin Ketteler

**Affiliations:** MRC Laboratory for Molecular Cell Biology, University College London, London WC1E 6BT, United Kingdom

**Keywords:** autophagy, cysteine protease, deubiquitylation (deubiquitination), post-translational modification, ubiquitin-conjugating enzyme (E2 enzyme), ATG4B, Atg8, deconjugation, GABARAPL2, LC3ylation

## Abstract

Microtubule-associated protein 1 light chain 3 α (LC3)/GABA type A receptor-associated protein (GABARAP) comprises a family of ubiquitin-like proteins involved in (macro)autophagy, an important intracellular degradation pathway that delivers cytoplasmic material to lysosomes via double-membrane vesicles called autophagosomes. The only currently known cellular molecules covalently modified by LC3/GABARAP are membrane phospholipids such as phosphatidylethanolamine in the autophagosome membrane. Autophagy-related 4 cysteine peptidase (ATG4) proteases process inactive pro-LC3/GABARAP before lipidation, and the same proteases can also deconjugate LC3/GABARAP from lipids. To determine whether LC3/GABARAP has other molecular targets, here we generated a pre-processed LC3B mutant (Q116P) that is resistant to ATG4-mediated deconjugation. Upon expression in human cells and when assessed by immunoblotting under reducing and denaturing conditions, deconjugation-resistant LC3B accumulated in multiple forms and at much higher molecular weights than free LC3B. We observed a similar accumulation when pre-processed versions of all mammalian LC3/GABARAP isoforms were expressed in ATG4-deficient cell lines, suggesting that LC3/GABARAP can attach also to other larger molecules. We identified ATG3, the E2-like enzyme involved in LC3/GABARAP lipidation, as one target of conjugation with multiple copies of LC3/GABARAP. We show that LC3B–ATG3 conjugates are distinct from the LC3B–ATG3 thioester intermediate formed before lipidation, and we biochemically demonstrate that ATG4B can cleave LC3B–ATG3 conjugates. Finally, we determined ATG3 residue Lys-243 as an LC3B modification site. Overall, we provide the first cellular evidence that mammalian LC3/GABARAP post-translationally modifies proteins akin to ubiquitination (“LC3ylation”), with ATG4 proteases acting like deubiquitinating enzymes to counteract this modification (“deLC3ylation”).

## Introduction

Protein degradation in eukaryotic cells occurs via two main pathways: macroautophagy (hereafter autophagy) and the ubiquitin–proteasome system (UPS).^3^ Autophagy involves the trafficking of bulk cytoplasmic material to lysosomes via autophagosomes (double-membrane–bound organelles) (1) and can degrade entire organelles, protein aggregates, and long-lived proteins. In contrast, the UPS targets individual polypeptides for proteolysis as signaled by the small protein ubiquitin (2). Ubiquitin becomes covalently attached via its C terminus to the lysine or amino group of target proteins, forming a stable amide linkage and acting as a post-translational modification. This reaction occurs via ATP-dependent activation of ubiquitin by E1-activating enzymes (3), followed by the action of E2-conjugating enzymes and E3 ligase components (4). The attachment of polyubiquitin chains linked via their Lys-48 residues is the canonical signal for proteasome-mediated degradation of the target protein, whereas other types of ubiquitination modification exist that can have multiple cellular consequences (5). Ubiquitination is reversed by deubiquitinating enzymes (DUBs), a set of cysteine proteases and metalloproteases that can cleave the amide bond between ubiquitin and its targets (6).

Autophagy is regulated by two consecutive ubiquitin-like conjugation reactions that closely resemble ubiquitination, both in terms of their biochemical mechanism and involvement of proteins that bear the ubiquitin β-grasp fold within their tertiary structure (7). The first is the ATG12–ATG5 conjugation system, in which the ubiquitin-like protein ATG12 is covalently attached to ATG5 by E1-like ATG7 and E2-like ATG10 (8, 9). The stable ATG12–ATG5 conjugate associates with ATG16L1 protein, forming a multimeric complex that localizes to the outer membrane of forming autophagosomes and dissociates prior to their closure (10–12). The second ubiquitin-like reaction is the LC3–PE conjugation system, involving covalent attachment of small (∼13–17 kDa) ubiquitin-like LC3/GABARAP proteins (ATG8 in yeast) to phospholipids on the autophagosome membrane such as phosphatidylethanolamine (PE) (13–15). This reaction known as LC3 lipidation is mediated by the same E1-like enzyme (ATG7), a distinct E2-like enzyme (ATG3), and enhanced in an E3-like manner by the ATG12–ATG5 conjugate (13, 16–18). LC3 lipidation functions in several aspects of autophagy, including membrane expansion, cargo recruitment, and autophagosome–lysosome fusion (19–24).

Unlike ATG12–ATG5 conjugation, LC3/GABARAP lipidation can be reversed by the DUB-like cysteine protease ATG4 in a reaction known as delipidation or deconjugation (25, 26). ATG4 also performs an upstream processing event known as LC3/GABARAP priming, in which a C-terminal extension of one or more amino acid residues is rapidly removed from newly-synthesized pro-LC3/GABARAP through proteolysis (14, 25, 26). This step is essential for subsequent lipidation because it exposes the C terminus of the active glycine residue in LC3/GABARAP that later becomes attached to PE via an amide bond (13, 25).

In humans, there are at least seven isoforms of LC3/GABARAP divided into the LC3 subfamily (MAP1LC3A, -B, -B2, and -C) and the GABARAP subfamily (GABARAP, -L1, and -L2) (16, 27–31), of which LC3B is the most studied and widely utilized as a marker for autophagosomes in mammalian cells. Lipidated LC3B localizes to autophagosomes during their formation and remains associated following autophagosome–lysosome fusion, eventually being degraded or recycled (14, 32). The conversion of primed LC3B (LC3B-I) to the lipidated form (LC3B-II) can also be monitored by Western blotting because the lipidated form is more negatively charged and thus migrates slightly faster in SDS-PAGE (14, 33). Four mammalian ATG4 proteases (ATG4A, -B, -C, and -D) process the LC3/GABARAP isoforms with partially overlapping redundancies, with ATG4B being the functionally dominant isoform in autophagy and essential for priming LC3B (34–38).

Whereas ubiquitin is reported to attach to thousands of cellular target proteins (39), the only known target molecules of LC3/GABARAP conjugation are phospholipids, including PE and possibly also phosphatidylserine (13, 15, 40). Considering LC3/GABARAP conjugation closely resembles ubiquitination and ATG4 is DUB-like, we decided to investigate whether other cellular targets might exist that can be modified with LC3/GABARAPs (“LC3ylation”) and deconjugated by ATG4 (“deLC3ylation”).

## Results

### Deconjugation-resistant LC3B reveals presence of high molecular weight LC3B conjugates in cells

**Figure 1.**
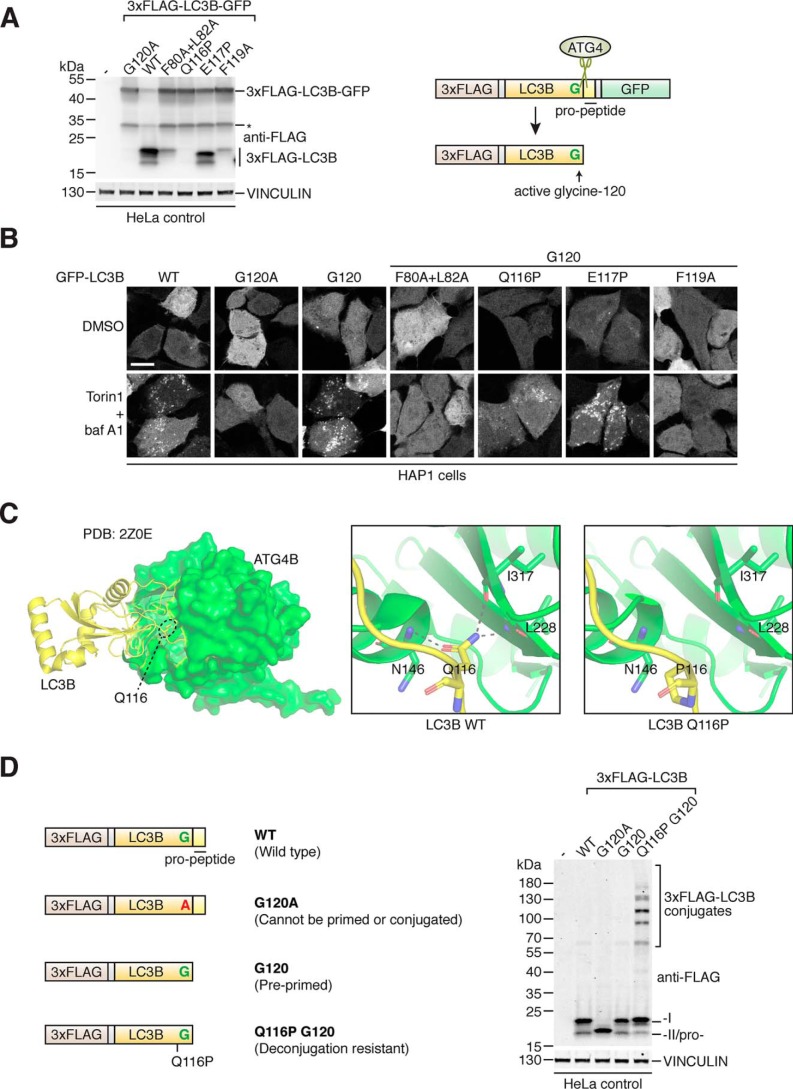
**Development of deconjugation-resistant LC3B mutant reveals novel LC3B conjugates in cells.** *A,* LC3B priming assay in HeLa cells using transiently transfected 3xFLAG–LC3B–GFP construct and point mutants. Western blotting of lysates using anti-FLAG antibody is shown on the *left-hand side* (*asterisk* indicates nonspecific cleavage product) and schematic of construct processing by endogenous ATG4 in cells shown on *right. B,* confocal microscopy of GFP localization in HAP1 control cells transiently transfected with GFP–LC3B constructs and treated with DMSO or 250 nm Torin1 + 10 nm baf A1 for 3 h prior to fixation. Point mutations were introduced into the pre-primed G120 form of GFP–LC3B where indicated. *Scale bar,* 10 μm. *C,* location of LC3B Gln-116 residue within the binding pocket of ATG4B visualized using co-crystal structure PDB code 2Z0E (42), with LC3B displayed as a ribbon in *yellow* and ATG4B as surface model in *green* (*left-hand side*). *Boxes* show close up of H-bond interactions between LC3B Gln-116 and ATG4B residues Asn-146, Ile-317, and Leu-228 for WT (*middle*) and modeled Q116P mutation (*right*). *D,* 3xFLAG–LC3B constructs (schematics shown on *left*) were transiently expressed in HeLa control cells prior to lysis and Western blotting detection using anti-FLAG antibody (*right*).

As a tool to identify potential novel LC3/GABARAP conjugation targets, we decided to develop a deconjugation-resistant form of LC3B that could be expressed in mammalian cells. We sought to identify a mutation in LC3B that renders it resistant to processing by ATG4 proteases, but does not abolish conjugation when introduced into a pre-primed form of LC3B (LC3B G120). In the literature, we identified the F80A/L82A double mutant (41) and F119A (42) as mutations in LC3B known to impair cleavage by mammalian ATG4 proteases, although the effects of these mutations on lipidation are unknown. Because LC3B is structurally ubiquitin-like and studies have shown that ubiquitin and small ubiquitin-like modifiers can be rendered resistant to deconjugation by mutating a residue near their protease cleavage site to proline (43, 44), we also decided to generate two candidate equivalent LC3B mutations (Q116P and E117P). We introduced each of these mutations into a synthetic priming sensor construct consisting of full-length LC3B tagged at the C terminus with GFP and the N terminus with 3xFLAG. Expression of these constructs allowed us to compare the effect of each mutation on processing by endogenous ATG4 proteases in human HeLa cells. As seen in Fig. 1*A*, all tested mutations impeded ATG4-mediated processing to varying degrees compared with WT LC3B, based on an increase in the level of unprocessed 3xFLAG–LC3B–GFP and a reduction in the level of processed 3xFLAG–LC3B. The Q116P mutation in particular led to the most robust reduction in processing, comparable with the negative control G120A mutant that cannot be primed or lipidated (14).

To test whether the LC3B mutations were compatible with PE conjugation, we introduced them into pre-primed GFP–LC3B G120 that localizes to distinct cytoplasmic puncta corresponding to autophagosomes upon lipidation (15). We expressed these constructs in human HAP1 cells and monitored localization following co-treatment with the mechanistic target of rapamycin inhibitor Torin1 (45) and lysosomal inhibitor bafilomycin A_1_ (baf A1) to induce autophagosome accumulation. As seen in Fig. 1*B*, the LC3B mutants F80A/L82A and F119A showed a defect in autophagosome localization similar to G120A, suggesting these mutations impaired lipid conjugation. In contrast, Q116P and E117P could localize to puncta in response to treatment, similar to LC3B WT and G120, suggesting these mutations are compatible with conjugation. Overall, the Q116P mutation best fit the criteria of impairing ATG4-mediated processing while permitting conjugation, making it suitable for generating a deconjugation-resistant LC3B mutant. Using the published LC3B–ATG4B co-crystal structure (42), we determined that the side chain of LC3B residue Gln-116 forms three hydrogen bonds with the backbone of ATG4B (at residue positions Ile-317, Asn-146, and Leu-228), which are lost in the LC3B Q116P mutant, suggesting these interactions are important for ATG4B-mediated processing (Fig. 1*C*).

To determine the effect of impaired LC3B deconjugation in cells, we expressed 3x-FLAG–tagged deconjugation-resistant LC3B Q116P G120 in WT HeLa cells, with specific detection by Western blotting using an anti-FLAG antibody. As seen in Fig. 1*D*, LC3B Q116P G120 could be converted to the -II form similar to LC3B WT and G120. In contrast, LC3B G120A was expressed as a single band corresponding to the pro-form (46). Strikingly, at much higher molecular weights than LC3B-I and LC3B-II, we could observe the presence of multiple bands that were specifically enriched in cells expressing deconjugation-resistant LC3B Q116P G120. These bands ranged from an apparent molecular mass of ∼60 kDa to over 180 kDa and were not affected by autophagy induced by serum and amino acid starvation (Fig. S1*A*) or pharmacological treatments (Fig. S1*B*). Because Western blotting was performed under reducing and denaturing conditions, the additional bands are unlikely to represent noncovalent and redox-sensitive protein interactions with LC3B (47). Therefore, our data indicate that LC3B can covalently conjugate to other unknown molecules in the cell that would normally be substrates of ATG4-mediated deconjugation.

### ATG4 proteases are responsible for counteracting LC3/GABARAP conjugate formation in cells

**Figure 2.**
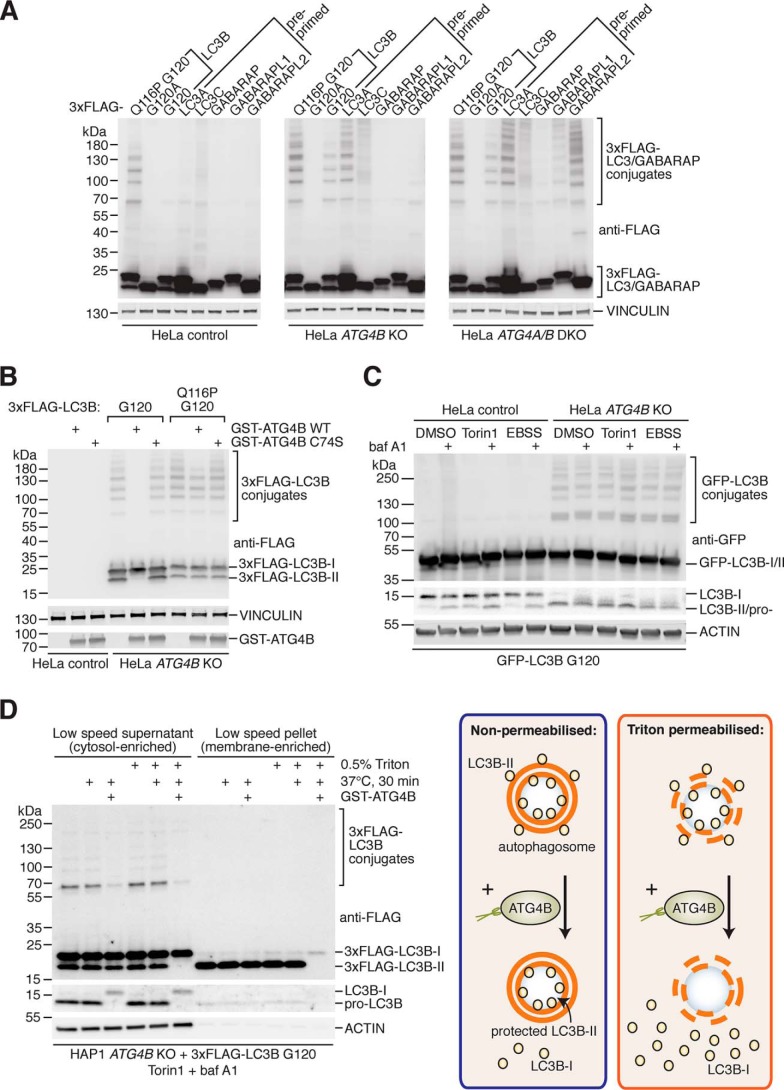
**Identification and characterization of LC3/GABARAP conjugates in ATG4-deficient cells.** *A,* expression of pre-primed 3xFLAG-tagged LC3/GABARAP isoforms (LC3A G120, LC3C G126, GABARAP G116, GABARAPL1 G116, and GABARAPL2 G116) and LC3B mutants in control, *ATG4B* KO, and *ATG4A/B* DKO HeLa cells using transient transfection prior to Western blot analysis. Panels are vertically divided for presentation; all results are from the same membrane and displayed at the same exposure. *B,* digestion of 3xFLAG–LC3B conjugates from HeLa *ATG4B* KO cells. Lysates were prepared in IP buffer and subsequently incubated on ice (untreated samples) or at 37 °C for 1 h with purified recombinant GST–ATG4B WT or inactive (C74S) mutant at a final concentration of 0.02 mg/ml, prior to Western blotting. ATG4B antibody was used to detect GST–ATG4B. *C,* assessment of GFP–LC3B conjugates following autophagy modulation. HeLa control and *ATG4B* KO cells stably expressing GFP–LC3B G120 were treated with combinations of EBSS, DMSO, 250 nm Torin1, and 10 nm baf A1 for 3 h prior to lysis and Western blot analysis. Endogenous LC3B was detected using anti-LC3B antibody, and GFP–LC3B conjugates were detected using anti-GFP. *D,* cell fractionation and GST–ATG4B protease protection assessment of 3xFLAG–LC3B conjugates. HAP1 *ATG4B* KO cells transiently transfected with 3xFLAG–LC3B G120 were treated for 3 h with 250 nm Torin1 and 10 nm baf A1 prior to sample preparation, processing, and analysis by Western blotting (*left*). 3xFLAG–LC3B G120 was detected using anti-FLAG antibody, and endogenous LC3B was detected using anti-LC3B (consisting of pro-LC3B in untreated samples). On *right* is a schematic highlighting how the presence of detergent (Triton) disrupts protease protection as a positive control.

To distinguish whether high molecular weight LC3B conjugates accumulated in cells due to loss of ATG4-mediated deconjugation rather than a secondary effect of Q116P mutation, we assessed whether genetic deficiency of ATG4 proteases induced conjugate accumulation. We also sought to determine whether other mammalian LC3/GABARAP isoforms had the ability to form conjugates. Using WT HeLa cells *versus* those lacking *ATG4B* or *ATG4A* and *ATG4B*, we expressed LC3B G120 and the equivalent pre-primed form of the other five conserved LC3/GABARAP isoforms (LC3A, LC3C, GABARAP, GABARAPL1, and GABARAPL2), alongside LC3B Q116P G120 as a positive control and LC3B G120A as a negative control (Fig. 2*A*). We found that indeed the expression of LC3B G120 in *ATG4B* KO cells resulted in the accumulation of conjugates with a strikingly similar pattern to the Q116P G120 mutant, suggesting that LC3B conjugates accumulate as a result of impaired ATG4B-mediated deconjugation. The lack of conjugate formation for LC3B G120A demonstrates that priming and thus C-terminal Gly-120 exposure is required for conjugate formation. We found that conjugates of pre-primed LC3A and LC3C also accumulated when expressed in ATG4B-deficient cells, consistent with ATG4B being the main ATG4 isoform involved in LC3 subfamily processing (37). Some putative conjugates were faintly visible in control HeLa cells, particularly upon expression of pre-primed 3xFLAG-LC3C, raising the possibility that LC3/GABARAP conjugates may exist at a reduced level under more physiological conditions when ATG4 is expressed. Pre-primed GABARAP subfamily proteins were relatively poor at forming conjugates when expressed in *ATG4B* KO cells. Remarkably, we could observe dramatic GABARAP subfamily conjugate accumulation in cells lacking both ATG4A and ATG4B, suggesting that ATG4A is involved in counteracting the formation of GABARAP subfamily conjugates and consistent with the known ability of ATG4A to prime GABARAP subfamily proteins (35, 37). Interestingly, all tested LC3/GABARAP isoforms could form conjugates in this cell model with a very similar band pattern, suggesting they likely modify the same target molecules (Fig. 2*A*).

Next, we determined whether LC3/GABARAP conjugates were direct substrates of ATG4. We treated lysates from *ATG4B* KO cells expressing pre-primed LC3B G120 and deconjugation-resistant LC3B Q116P G120 with purified recombinant GST-tagged ATG4B or catalytic inactive mutant C74S as a negative control (Fig. 2*B*). This revealed that 3xFLAG–LC3B G120 conjugates were indeed biochemical substrates of ATG4B, because their detection was lost upon treatment with active ATG4B WT but not inactive ATG4B C74S. As an internal positive control, lipidated 3xFLAG–LC3B G120 was efficiently cleaved by recombinant ATG4B WT. This experiment also demonstrated that 3xFLAG–LC3B Q116P G120 conjugates and the corresponding lipidated form were resistant to ATGB activity *in vitro*. Overall, these results confirm the enzymatic role of ATG4 proteases in counteracting LC3/GABARAP conjugate formation in cells.

### LC3B conjugates in ATG4B KO cells are not autophagy substrates

Considering LC3B has a well-established role in autophagy, we decided to investigate whether LC3B conjugates were also affected by treatments that modulate LC3B lipidation and autophagy. As seen in Fig. 2*C*, we could observe GFP–LC3B conjugate accumulation in HeLa *ATG4B* KO cells stably expressing GFP–LC3B G120. We noted that the overall level of GFP–LC3B conjugates in *ATG4B* KO cells appeared unaffected by autophagy-modulating treatments (Fig. 2*C*), consistent with our results for Q116P conjugates (Fig. S1). This suggests that GFP–LC3B conjugates accumulate stably in the absence of ATG4B-mediated deconjugation and are not degraded by autophagy, especially considering that autophagy is known to be functional in *ATG4B* KO cells rescued with GFP–LC3B G120 (38). We could also observe the formation of GFP–LC3B G120 conjugates in *ATG4A/B* DKO cells when expressed at levels close to that of endogenous LC3B, ruling out the possibility that LC3/GABARAP conjugates are an overexpression artifact (Fig. S2).

Next, in a cell fractionation and protease protection experiment, we observed that 3xFLAG–LC3B G120 could form conjugates when expressed in HAP1 *ATG4B* KO cells, demonstrating that this phenomenon is not restricted to a single-cell model (Fig. 2*D*). The conjugates were observed to be present in the soluble fraction but absent from the membrane fraction enriched in 3xFLAG–LC3B-II, suggesting that they were not lipidated. Furthermore, 3xFLAG–LC3B conjugates were not protected from digestion by purified recombinant GST–ATG4B in the absence of detergent, suggesting they were not located within the sealed membrane organelles such as autophagosomes.

### ATG3 is a protein target of LC3/GABARAP conjugation and ATG4-mediated deconjugation

We took a candidate-based approach to identify novel targets of LC3B conjugation, prioritizing reported LC3B-interacting proteins (48) that are predicted to be soluble, cytoplasmic, and not known to be degraded by autophagy. We also considered the minimum molecular mass of LC3B targets observed by Western blotting, which we predicted to be ∼35 kDa after accounting for the mass of tagged LC3B (Fig. 2*D*). The strongest candidate based on these criteria was ATG3, the soluble cytoplasmic E2-like conjugating enzyme component of the LC3/GABARAP lipidation reaction, which has a predicted molecular mass of 35.8 kDa (17, 49).

**Figure 3.**
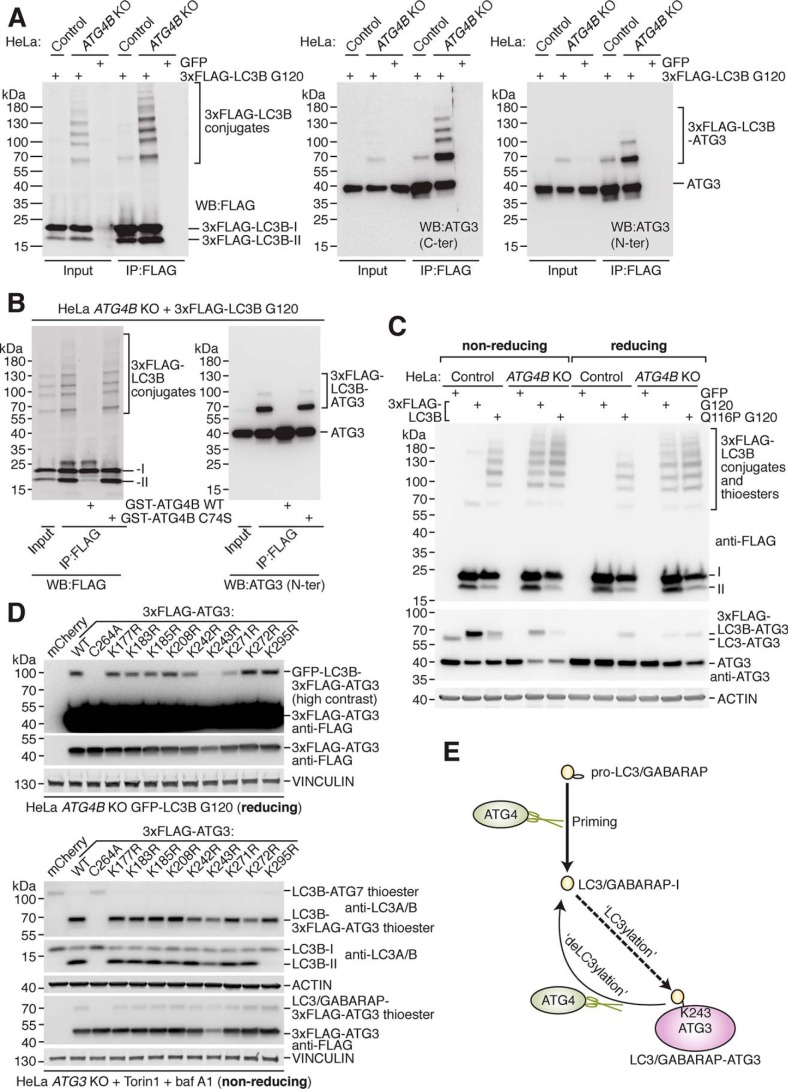
**ATG3 protein is a target of LC3/GABARAP conjugation and ATG4-mediated deconjugation.** *A,* enrichment of transiently expressed 3xFLAG–LC3B G120 from HeLa control and *ATG4B* KO cells by IP with anti-FLAG antibody prior to Western blotting with anti-FLAG antibody (*left*) and anti-ATG3 antibodies (*middle, right panel*). Cells were transfected with GFP as a negative control. *B,* 3xFLAG–LC3B G120 expressed in HeLa *ATG4B* KO cells was enriched by IP with anti-FLAG antibody prior to treatment with 0.02 mg/ml recombinant GST–ATG4B WT or C74S at 37 °C for 1 h and Western blotting using anti-FLAG and anti-ATG3 N-ter antibody. *C,* HeLa control and *ATG4B* KO cells were transfected with 3xFLAG–LC3B G120, 3xFLAG–LC3B Q116P G120, or GFP as a negative control prior to harvesting. Western blotting was performed to detect 3xFLAG–LC3B and ATG3 using the same lysates diluted with either nonreducing (β-mercaptoethanol excluded) or reducing (standard recipe) sample buffer. Thioester-linked species are detected under nonreducing conditions only. *D,* Western blotting of HeLa *ATG4B* KO GFP–LC3B G120 cells transiently transfected with 3xFLAG–ATG3 WT and point mutant constructs. Samples were lysed in buffer lacking NEM and processed under reducing conditions (*upper panel*). The same constructs were used to rescue HeLa *ATG3* KO cells treated for 3 h with 250 nm Torin1 and 10 nm baf A1 and assessed by Western blotting under nonreducing conditions (*lower panel*). *E,* schematic of human ATG4 protease cellular function in protein deconjugation (*deLC3ylation*), which counteracts covalent attachment of LC3/GABARAP (LC3ylatio*n*) to ATG3 protein at residue Lys-243.

To determine whether ATG3 is a target of LC3B conjugation, we enriched 3xFLAG–LC3B G120 conjugates from HeLa control and *ATG4B* KO cells by immunoprecipitation (IP) (Fig. 3*A*). Blotting with anti-FLAG antibody confirmed the enrichment of 3xFLAG–LC3B G120 and conjugates in the IP *versus* input lysates. We probed the same samples with two different antibodies targeting different portions of endogenous ATG3 (C terminus and N terminus, respectively). In addition to full-length ATG3, both antibodies specifically detected a number of high molecular weight conjugate bands likely corresponding to 3xFLAG–LC3B–ATG3 (Fig. 3*A*). We then digested 3xFLAG–LC3B conjugates enriched from *ATG4B* KO cells by IP prior to blotting for FLAG and ATG3. As seen in Fig. 3*B*, high molecular weight bands detected using ATG3 antibody were specifically lost upon digestion with active GST–ATG4B, confirming their identity as 3xFLAG–LC3B conjugated to ATG3.

It is well-established that ATG3 forms a thioester-linked covalent intermediate with the C terminus of LC3/GABARAP during the PE conjugation reaction in WT cells (17), but this species can only be detected in lysates under nonreducing conditions (47). By comparing the same lysates prepared under nonreducing and reducing conditions, we confirmed that 3xFLAG–LC3B–ATG3 and conjugates formed by deconjugation-resistant LC3B or pre-primed LC3B in HeLa *ATG4B* KO cells are extremely stable under reducing conditions, making them distinguishable from the thioester-linked species in WT cells that are fully resolved under reducing conditions (Fig. 3*C*).

Finally, we tested lysine residues by mutagenesis to identify a target residue of LC3B modification on ATG3. We specifically reconstituted the modification of transiently-expressed 3xFLAG–ATG3 in HeLa *ATG4B* KO cells stably expressing GFP–LC3B G120 (Fig. S3*A*). We found that ATG3 activity was important, because catalytically-inactive ATG3 C264A could not be efficiently modified (Fig. S3*A*). We therefore generated HeLa *ATG3* KO cells using CRISPR-Cas9 (validated by Western blotting in Fig. S3*B*) to test in parallel the function of putative 3xFLAG–ATG3 modification site mutants in restoring endogenous LC3B lipidation. This could help to rule out inactivity being the reason for loss of LC3B conjugation. We then generated 3xFLAG–ATG3 constructs in which all or different sets of lysine residues were mutated to arginine (Fig. S3*C*). The first 13 lysine residues in ATG3 were important for efficient ATG3 expression but dispensable for LC3B lipidation (Fig. S3, *D* and *E*). In contrast, ATG3 lacking the last nine lysines could not rescue LC3B lipidation nor be efficiently modified by LC3B (Fig. S3*D*), despite expressing well and forming a thioester with endogenous LC3/GABARAP (Fig. S3*E*). We therefore tested single mutants of the last nine ATG3 lysine residues, uncovering Lys-243 as specifically required for efficient GFP–LC3B modification but not ATG3 function, suggesting Lys-243 is a major site of covalent LC3B modification (Fig. 3*D*). Interestingly, we also found that Lys-295 was uniquely required for LC3B lipidation but not conjugate or thioester formation.

In conclusion, our study demonstrates that human ATG4 proteases function as deLC3ylation enzymes by counteracting the covalent attachment of LC3/GABARAP to other proteins in the cell such as residue Lys-243 of ATG3 (Fig. 3*E*).

## Discussion

Ubiquitin modifications play a major role in specifying protein fate and function, whereas LC3/GABARAP proteins have previously been believed to exclusively modify phospholipids to facilitate autophagy. We now demonstrate that LC3/GABARAP can modify other proteins, expanding our knowledge of the repertoire of post-translational modifications that mammalian cells can potentially utilize to modulate protein function. We also show that the proteases ATG4A and ATG4B have a novel deconjugation function, acting as DUB-like enzymes to reverse LC3/GABARAP protein modifications. It proved necessary to use cellular models in which ATG4 deconjugation activity is impaired to facilitate the discovery and characterization of LC3/GABARAP protein conjugates. Therefore, we speculate that the abundance of these protein conjugates is normally limited by ATG4 activity in the cytoplasm. Any scenarios in which ATG4 activity is modulated could potentially affect the level of LC3/GABARAP protein conjugates, although this requires further study. For example, ATG4 proteases are subject to regulation by post-translational modifications such as oxidation (50) and phosphorylation (51–53). These modifications are proposed to provide mechanisms for spatiotemporal control of ATG4 activity, and they could therefore be candidates for controlling the level of LC3/GABARAP protein conjugates in cells.

We found that one major site of conjugation by LC3B on ATG3 is Lys-243. ATG12 conjugation to ATG3 also occurs at Lys-243, catalyzed by ATG3 itself (54). LC3B–ATG3 modification may represent a similar autoconjugation mechanism due to its requirement for ATG3 activity. The ATG12–ATG3 conjugate is linked to mitochondrial homeostasis and late endosome function (55), while further studies are needed to elucidate the function of LC3/GABARAP conjugation to ATG3 and its deconjugation by ATG4 proteases. Indeed, it may now be necessary to distinguish whether previously-studied phenotypes of ATG3 K243R (54, 55) occur as a consequence of impaired ATG12 or LC3/GABARAP conjugation. Notably, in our pulldown experiments we observed multiple species of LC3B–ATG3 conjugates at distinct molecular weight intervals corresponding to the size of 3xFLAG–LC3B, suggesting other ATG3 or LC3B residues may be modified.

Multiple models of *ATG4* KO yeast cells expressing pre-primed ATG8 have been generated and studied extensively in the context of ATG4 delipidation (56–58). These could be utilized to study whether ATG4 has an evolutionarily conserved role in deconjugating ATG8 from proteins. Interestingly, yeast ATG8 can stably conjugate to ATG3 residue Ser-4 *in vitro* (59); however, this residue is not conserved in mammalian ATG3 (60).

Finally, our study has described the rational development of a deconjugation-resistant LC3B mutant (Q116P G120). We expect this to be an important research tool for investigating the roles of LC3B deconjugation in different contexts, given the emerging multifaceted functions of LC3/GABARAP proteins in pathways beyond canonical autophagy such as secretion (61) and LC3-associated phagocytosis (62).

## Experimental procedures

### Cell culture

HeLa control, *ATG4B* KO, and *ATG4A/B* DKO (clone 25) cells and lines stably expressing GFP–LC3B constructs under control of the *CMV* or *PGK* promoter were generated and validated in a previous study (38). These were grown in Dulbecco's modified Eagle's medium high glucose with GlutaMax containing 1 mm pyruvate, supplemented with penicillin/streptomycin (100 units/ml) and 10% FBS. HAP1 control cells (Horizon Genomics, C631) and HAP1 *ATG4B* KO cells (Horizon Genomics, HZGHC001241c011) were grown in Iscove's modified Dulbecco's medium containing 25 mm HEPES and l-glutamine supplemented with penicillin/streptomycin (100 units/ml each) and 10% heat-inactivated FBS. All medium and supplements were purchased from Thermo Fisher Scientific. Cells were maintained at 37 °C and 5% CO_2_.

### Plasmid transfection

HeLa cells and lines grown in 12-well plates (seeded at a density of 1 × 10^4^ cells per well) were transfected with 250 ng of total plasmid DNA using 1.5 μl of jetPRIME reagent (Polyplus transfection) and 50 μl of jetPRIME buffer per well (containing 1 ml of growth medium). Transfections were scaled up or down according to growth medium volume, and cell densities were scaled according to the well surface area. HAP1 cells were transfected using polyethyleneimine as described previously (protease protection experiment) (38) or by using Viromer Yellow (Lipocalyx GmbH) according to the manufacturer's protocol (microscopy experiment). All experiments were performed 24 h after transfection.

### CRISPR-Cas9 genome editing

Sanger CRISPR Finder (63) was used to select a specific spCas9 target sequence with no predicted exonic off-target sites in exon 5 of human *ATG3* (location: chr3:112,548,624–112,548,646; CRISPR ID: 959983715). Complementary oligonucleotides incorporating the 19-bp target sequence adjacent to the protospacer adjacent motif were designed and synthesized with cloning-compatible overhangs, consisting of sgATG3(+) (5′-CACCGTATAGTGCCGTGCTATAAG-3′) and sgATG3(−) (5′-AAACCTTATAGCACGGCACTATAC-3′). These oligonucleotides were annealed, phosphorylated, and cloned into BbsI-digested pSpCas9(BB)-2A-Puro (PX459) version 2.0, a gift from Feng Zhang (Addgene plasmid no. 62988), as described (64). WT HeLa cells were transfected with the resulting plasmid as described above for “plasmid transfection,” followed by 48 h of growth in the presence of 1 μg/ml puromycin. Surviving cells were then expanded into 10-cm dishes for 72 h, prior to seeding at a limiting dilution in 96-well plates. After 2 weeks, clonal cell lines were expanded into 12-well plates and screened for loss of ATG3 protein by Western blotting. One verified HeLa *ATG3* KO cell line (referred to as clone 31) was used for this study.

### Antibodies and reagents

Primary antibodies and dilutions used for Western blotting were as follows: ATG3 C-ter (1:1,000, Abcam, ab108282), ATG3 N-ter (1:1,000, Abcam, ab108251), ATG4B (1:1,000, Cell Signaling Technology, no. 5299), β-actin (1:4,000, Sigma, A1978), FLAG biotin-conjugated (1:1,000, Sigma, F9291), GFP (1:2,000, Clontech, no. 632381), LC3A/B (1:500, Cell Signaling Technology, no. 12741), LC3B (1:1,000, Sigma, L7543), SQSTM1 (1:1,000, Sigma, P0057), vinculin (1:1,000, Abcam, ab129002).

Secondary antibodies for Western blotting with chemiluminescence-based detection were as follows: goat anti-rabbit/anti-mouse IgG, HRP-linked (1:5,000, Cell Signaling Technology, no. 7074/no. 7076). For fluorescence-based detection, the following were used: IRDye 800CW goat anti-rabbit/anti-mouse (1:15,000 with 0.02% SDS, LI-COR no. 926-32211/926-32212); IRDye 680LT goat anti-rabbit/anti-mouse (1:25,000 with 0.02% SDS, LI-COR no. 926-68021/926-68020).

Torin1 was purchased from Merck-Millipore (no. 475991), and bafilomycin A_1_ from *Streptomyces griseus* was purchased from Sigma (B1793); both were dissolved in DMSO. Recombinant GST–ATG4B WT and C74S were produced and purified as described previously (65). Earle's balanced salt solution (EBSS) containing calcium and magnesium was purchased from Thermo Fisher Scientific (no. 24010-043).

### Plasmids and cloning

New mutations were introduced into the Gateway entry clones encoding LC3B WT and LC3B G120 containing stop codons described previously (38). LC3B F80A/L82A was generated by overlap extension PCR. LC3B F119A was generated by conventional PCR using mutagenic reverse primers. The final product of each of these PCRs was purified and recombined with pDONR223 using BP clonase II enzyme mix (Life Technologies, Inc.) (66). LC3B Q116P and E117P were generated by site-directed mutagenesis using Q5 site-directed mutagenesis kit (New England Biolabs) according to the manufacturer's protocol. Primers used for PCRs are listed in Table S1. To generate LC3B mutant entry clones lacking stop codons, mutant LC3B-coding sequences containing stop codons (detailed above) were PCR-amplified using the common primers LC3B forward (5′-GGGGACAAGTTTGTACAAAAAAGCAGGCTTAATGCCGTCGGAGAAGACC-3′) and LC3B WT reverse with no stop codon (5′-GGGGACCACTTTGTACAAGAAAGCTGGGTACACTGACAATTTCATCCCG-3′) prior to BP recombination of the PCR product with pDONR223.

To generate an ATG3 entry clone encoding WT ATG3 with a stop codon, the ATG3 CDS was PCR-amplified from pDONR223_ATG3_WT_V5 (a gift from Jesse Boehm, Matthew Meyerson, and David Root, Addgene plasmid no. 82952 (67)) using the primers ATG3 forward (5′-GGGGACAAGTTTGTACAAAAAAGCAGGCTTAATGCAGAATGTGATTAATACTGTG-3′) and ATG3 reverse with stop codon (5′-GGGGACCACTTTGTACAAGAAAGCTGGGTATTACATTGTGAAGTGTCTTGTGTAG-3′). The same primers were also used to amplify a custom synthetic dsDNA fragment (GeneStrands, Eurofins Genomics) encoding full-length ATG3 CDS with all lysine residues converted to arginine (allKR), achieved by mutating all in-frame AAA or AAG codons to AGA or AGG, respectively. PCR products were purified prior to BP recombination with pDONR223 to generate entry clones.

Tagged expression constructs of LC3B mutants and ATG3 WT/allKR were generated by recombining entry clones (above) with destination vectors described previously (38), using the LR clonase II enzyme mix. For 3xFLAG–LC3B–GFP constructs, entry clones lacking stop codons were recombined with pDEST–CMV–3xFLAG–gateway–EGFP. To make GFP–LC3B constructs, entry clones with stop codons were recombined with pDEST–CMV–N-EGFP. For 3xFLAG–LC3B constructs and 3xFLAG–ATG3, entry clones containing stop codons were recombined with the destination vector pCMV–tripleFLAG–Gateway (68). Further individual mutations were introduced into 3xFLAG–ATG3 using mutagenic primers listed in Table S2 and Q5 mutagenesis kit, or an equivalent inverse PCR method employing Phusion polymerase, DpnI digestion of the PCR mixture, followed by co-treatment with T4 polynucleotide kinase and T4 DNA ligase (all purchased from New England Biolabs). The hybrid 3xFLAG–ATG3 WT/allKR constructs 3xFLAG–ATG3 1–13KR and 14–22KR were generated by restriction-based cloning: 3xFLAG–ATG3 WT and 3xFLAG–ATG3 allKR were both digested using KpnI + PstI, with the resulting insert fragments (containing the sequence encoding the first 13 lysines or replaced arginines residues in ATG3) swapped between the two vectors. 3xFLAG-LC3/GABARAP isoforms (G116, G120, or G126 pre-primed versions) and negative control (LC3B G120A) were generated and described previously (38). As transfection controls, pEAK13–EGFP was used for expression of GFP alone (69), and pCMV SPORT6-mCherry was used for the expression of mCherry (52). Plasmids were validated by restriction enzyme digests and Sanger sequencing.

### Western blotting

Cells were washed in cold PBS prior to lysis on ice using lysis buffer (150 mm NaCl, 1% IGEPAL CA-630, 50 mm Tris-HCl, pH 8.0, cOmplete^TM^ EDTA-free Protease Inhibitor Mixture, Roche Applied Science) supplemented with 20 mm *N*-ethylmaleimide (unless otherwise stated) to inhibit ATG4 activity in lysates (38). Lysates were cleared by centrifugation at 15,000 × *g* at 4 °C, and the resulting pellet was discarded. Total protein concentrations were determined using Pierce BCA protein assay kit (Thermo Fisher Scientific), and lysates were diluted to approximately equal concentrations before addition of 5× SDS sample buffer (250 mm Tris-Cl, pH 6.8, 10% SDS, 50% glycerol, 25% β-mercaptoethanol, and 0.05% bromphenol blue) with immediate boiling at 95 °C for 5 min. When comparing nonreducing and reducing sample conditions, NEM was excluded from the lysis buffer, and lysates were divided into 2 equal volumes prior to addition of 3× SDS sample buffer (150 mm Tris-Cl, pH 6.8, 6% SDS, 30% glycerol, 15% β-mercaptoethanol, and 0.03% bromphenol blue), with β-mercaptoethanol added fresh for reducing sample buffer or substituted with distilled water for nonreducing sample buffer. Reduced samples were boiled as normal, and the nonreduced samples were kept at room temperature. Equal volumes of sample (typically 15–25 μg of total protein) were loaded into lanes of a 15-well 4–20% Mini-PROTEAN TGX Precast gel or a 26-well 4–20% Criterion TGX gel (Bio-Rad) and run at 115 V constant for 75 min or 200 V for 35 min. Protein was transferred to Immobilon-FL polyvinylidene difluoride membrane (Merck-Millipore) before blocking with 5% skimmed milk in PBS-T (PBS with 0.05% Tween 20) for 1 h. The membrane was probed with primary antibody in blocking buffer at room temperature for 1 h (for FLAG, actin, or vinculin antibodies) or overnight at 4 °C (for all other antibodies). After washing with PBS-T, the membrane was incubated in secondary antibody in blocking buffer for 1 h at room temperature. Washes were performed prior to image acquisition on an Odyssey IR imaging system (LI-COR Biosciences) or development using HRP substrate (EZ-ECL chemiluminescence detection kit for HRP, Geneflow Ltd.) followed by exposure on an ImageQuant chemiluminescent imaging system (GE Healthcare). Actin and vinculin were detected as loading controls. All experimental observations were confirmed in a minimum of three independent experiments.

### Cell fractionation and ATG4B protease protection assay

HAP1 *ATG4B* KO cells grown in a 10-cm tissue culture dish were transfected with 10 μg of plasmid DNA encoding 3xFLAG–LC3B G120. The next day, cells were treated for 3 h with 250 nm Torin1 and 10 nm baf A1 to induce lipidation of 3xFLAG–LC3B G120. Intact cells were then collected as a pellet by trypsinization, trypsin inactivation in complete growth medium, and centrifugation at 300 × *g* for 5 min. The cell pellet was washed once in cold PBS before resuspending in 0.7 ml of homogenization buffer (HB) consisting of 10 mm HEPES-KOH, pH 7.5, 0.22 m d-mannitol, 0.07 m sucrose, and cOmplete^TM^ EDTA-free protease inhibitor mixture, Roche Applied Science. Cells were ruptured by passing the suspension 10 times through a 27-gauge needle attached to a 1-ml syringe. The resulting homogenate was centrifuged twice at 300 × *g*, 4 °C, for 10 min, each time discarding the nuclear pellet. The post-nuclear supernatant was centrifuged at 15,200 × *g*, 4 °C, for 10 min to generate a membrane-enriched low-speed pellet that was resuspended in 0.7 ml of HB, while the cytosol-enriched supernatant was transferred to a new tube. The two fractions were then further divided into nonpermeabilized and permeabilized stocks by addition of 1/10 volume of HB or HB containing 5% Triton X-100. At this point, 1 m DTT was added to a final concentration of 4.5 mm. These stocks were then subdivided into reaction tubes containing a final concentration of 0.03 mg/ml GST–ATG4B or an equivalent volume of HB, and treated for 30 min at 37 °C, with one untreated sample kept on ice as a control. To stop the reactions, all tubes were placed on ice, and 5× SDS sample buffer was added prior to immediate boiling at 95 °C for 10 min. Equal volumes of sample were loaded on polyacrylamide gels and analyzed by Western blotting.

### Immunoprecipitation

HeLa cell lines grown in 10-cm dishes were transfected with a total of 2.5 μg of plasmid DNA. After 24 h, cells were washed in cold PBS and lysed in ∼1 ml of IP buffer (50 mm Tris-HCl, pH 8, 150 mm NaCl, 1 mm EDTA, 1% Triton X-100, and cOmplete^TM^ EDTA-free Protease Inhibitor Mixture, Roche Applied Science). Lysates were cleared by centrifugation at 15,000 × *g* for 10 min at 4 °C. After 100 μl input sample was collected into a separate tube and boiled in sample buffer, and the remaining cleared lysate was loaded onto anti-FLAG® M2 Affinity Gel (A2220, Sigma) that had previously been equilibrated by washing three times with 1 ml of IP buffer. Approximately 30 μl of packed gel was used per sample, and binding was performed at 4 °C for 3 h on a tube rotator. Gel was washed three times with 1 ml of IP buffer, and bound protein was eluted from the gel by incubating with 3xFLAG peptide (F4799, Sigma) at 150 ng/μl in IP buffer for 30 min at 4 °C with shaking. Eluted sample (∼100 μl) was carefully removed from pelleted gel prior to digestion with recombinant GST–ATG4B and/or boiling in 5× SDS sample buffer to prepare samples for Western blotting.

### Light microscopy

Cells seeded on borosilicate glass coverslips (VWR, 13 mm diameter, thickness no. 1.5) were washed in PBS and fixed for 10 min at room temperature in 4% paraformaldehyde in PBS. The coverslips were then washed, stained with 1 μg/ml Hoechst 33342 for 10 min, washed and mounted onto glass slides using ProLong Diamond mounting medium (Life Technologies, Inc.), and cured overnight at room temperature in the dark. Fixed cells were imaged on a Leica SP8 confocal laser-scanning microscope using a ×63 oil immersion objective with a numerical aperture (N.A.) of 1.4 at 400 Hz with sequential channel acquisition. Laser and gain settings were optimized for the brightest sample to avoid signal saturation, and the same settings were used to image all samples within a single experiment. All images shown are of a single confocal Z-slice.

### Protein structure modeling and visualization

The FASTA sequence for the ATG4B-LC3(1–124) (PDB code 2Z0E) complex was downloaded from PDB (Protein Data Bank). The LC3 residue Gln-116 was mutated to proline and submitted to SWISS-MODEL (70) for protein tertiary structure prediction using PDB code 2Z0E as a template. PyMOL was used for model visualization and reported an all atom root mean square deviation of 0.058 Å between the mutated model and actual crystal structure.

## Author contributions

A. A. and R. K. conceptualization; A. A. and R. K. resources; A. A., L. v. C., H. O., and T. S. data curation; A. A. and R. K. supervision; A. A., L. v. C., H. O., and K. K. validation; A. A., L. v. C., H. O., K. K., and T. S. investigation; A. A. and T. S. visualization; A. A., L. v. C., H. O., K. K., T. S., and R. K. methodology; A. A. writing-original draft; A. A. and R. K. project administration; A. A., T. S., and R. K. writing-review and editing; T. S. software; R. K. funding acquisition.

## Supplementary Material

Supporting Information

## Footnotes

3 The abbreviations used are:

UPS: ubiquitin–proteasome system
DUB: deubiquitinating enzyme
LC3: light chain 3
GABARAP: GABA type A receptor-associated protein
PDB: Protein Data Bank
FBS: fetal bovine serum
HRP: horseradish peroxidase
PE: phosphatidylethanolamine
EBSS: Earle's balanced salt solution
IP: immunoprecipitation
DKO: double knockout
NEM: *N*-ethylmaleimide
EGFP: enhanced green fluorescent protein
baf A1: bafilomycin A_1_.
