# Supplementary material for: Human ATG4 autophagy proteases counteract attachment of ubiquitin-like LC3/GABARAP proteins to other cellular proteins
Source: J Biol Chem. 2019 Jul 17;294(34):12610–21. doi: 10.1074/jbc.AC119.009977 (PMC6709618; doi:10.1074/jbc.AC119.009977)
Supplement: Supporting Information [file supp_294_34_12610__index.html]

Human ATG4 autophagy proteases counteract attachment of ubiquitin-like LC3/GABARAP proteins to other cellular proteins — ATG4 deconjugates LC3/GABARAP from proteins in human cells — Human ATG4 autophagy proteases counteract attachment of ubiquitin-like LC3/GABARAP proteins to other cellular proteins — ACCELERATED COMMUNICATION: ATG4 removes LC3 from proteins — Supporting Information 

# Human ATG4 autophagy proteases counteract attachment of ubiquitin-like LC3/GABARAP proteins to other cellular proteins

## Supporting Information

- Supporting Information (to be published online) - Supporting Information (Experimental procedures, Table S1-S2 and Fig S1-S3)
