# Supplementary material for: Human ATG4 autophagy proteases counteract attachment of ubiquitin-like LC3/GABARAP proteins to other cellular proteins
Source: J Biol Chem. 2019 Jul 17;294(34):12610–21. doi: 10.1074/jbc.AC119.009977 (PMC6709618; doi:10.1074/jbc.AC119.009977)
Supplement: Supporting Information [file supp_AC119.009977_153980_0_supp_354634_p7ss7p.pdf]

Table S1. Sequences of primers used to generate Gateway entry clones for LC3B mutants, using either LC3B WT or LC3B G120 as a template.

LC3B mutation	PCR method	Forward primer sequence (5' to 3')	Reverse primer sequence (5' to 3')
F80A+L82A	Overlap extension PCR (step 1 of 2: 5' fragment)	GGGGACAAGTTTGTACAAAAAAGCAGGCTTAATGCCGTCGGAGAAGACC	GTCCGTTCACCGCCAGGGCGAAGGCCTG
F80A+L82A	Overlap extension PCR (step 1 of 2: 3' fragment)	CAGGCCTTCGCCCTGGCGGTGAACGGAC	GGGGACCACTTTGTACAAGAAAGCTGGGTATTACACTGACAATTTCATCCCGAAC
F80A+L82A G120	Overlap extension PCR (step 1 of 2: 3' fragment)	CAGGCCTTCGCCCTGGCGGTGAACGGAC	GGGGACCACTTTGTACAAGAAAGCTGGGTATTACCCGAACGTCTCCTGGG
F80A+L82A	Overlap extension PCR (step 2 of 2: final product)	GGGGACAAGTTTGTACAAAAAAGCAGGCTTAATGCCGTCGGAGAAGACC	GGGGACCACTTTGTACAAGAAAGCTGGGTATTACACTGACAATTTCATCCCGAAC
F80A+L82A G120	Overlap extension PCR (step 2 of 2: final product)	GGGGACAAGTTTGTACAAAAAAGCAGGCTTAATGCCGTCGGAGAAGACC	GGGGACCACTTTGTACAAGAAAGCTGGGTATTACCCGAACGTCTCCTGGG
Q116P	Site-directed mutagenesis (NEB Q5 method)	TATGCCTCCCCGGAGACGTTC	GACCATGTACAGGAATCC
E117P	Site-directed mutagenesis (NEB Q5 method)	TGCCTCCCAGCCGACGTTCGGG	TAGACCATGTACAGGAATCC
F119A	Conventional PCR with mutagenic reverse primer	GGGGACAAGTTTGTACAAAAAAGCAGGCTTAATGCCGTCGGAGAAGACC	GGGGACCACTTTGTACAAGAAAGCTGGGTATTACACTGACAATTTCATCCCGGCCGTCTC
F119A G120	Conventional PCR with mutagenic reverse primer	GGGGACAAGTTTGTACAAAAAAGCAGGCTTAATGCCGTCGGAGAAGACC	GGGGACCACTTTGTACAAGAAAGCTGGGTATTACCCGGCCGTCTCCTGGG

Table S2. Sequences of primers used for 3xFLAG-ATG3 mutagenesis via inverse PCR (mutated nucleotide in lowercase).

**ATG3 mutation**	**Forward primer sequence (5' to 3')**	**Reverse primer sequence (5' to 3')**
C264A	AGTTCACCCAgcCAGGCATGCTG	GAACACATGGGAGGTGGT
K177R	GATACAAGGAgAATAGTAGAAGCTTG	TAGGGTAGCCTCATCTGT
K183R	GAAGCTTGTAgAGCCAAAACTG	TACTATTTTCCTTGTATCTAGG
K185R	TGTAAAGCCAgAACTGATGCTG	AGCTTCTACTATTTTCCTTG
K208R	ACTTATGATAgATATTACCAGACTC	GATGTAAAGGTCATAAGTTC
K242R	GATCATGTGAgGGAAAACAGTG	CTGACTGATGTCTTCATAC
K243R	CATGTGAAGAgAACAGTGACC	ATCCTGACTGATGTCTTC
K271R	GAGGTGATGAgGAAAATCATTGAGACTGTTG	AGCATGCCTGCATGGGTG
K272R	GTGATGAAGAgAATCATTGAGACTGTTGC	CTCAGCATGCCTGCATGG
K295R	ATTTTCTTGAgATTTGTACAAGCTG	AAGAAGATACATATGAACTCC

Figure S1

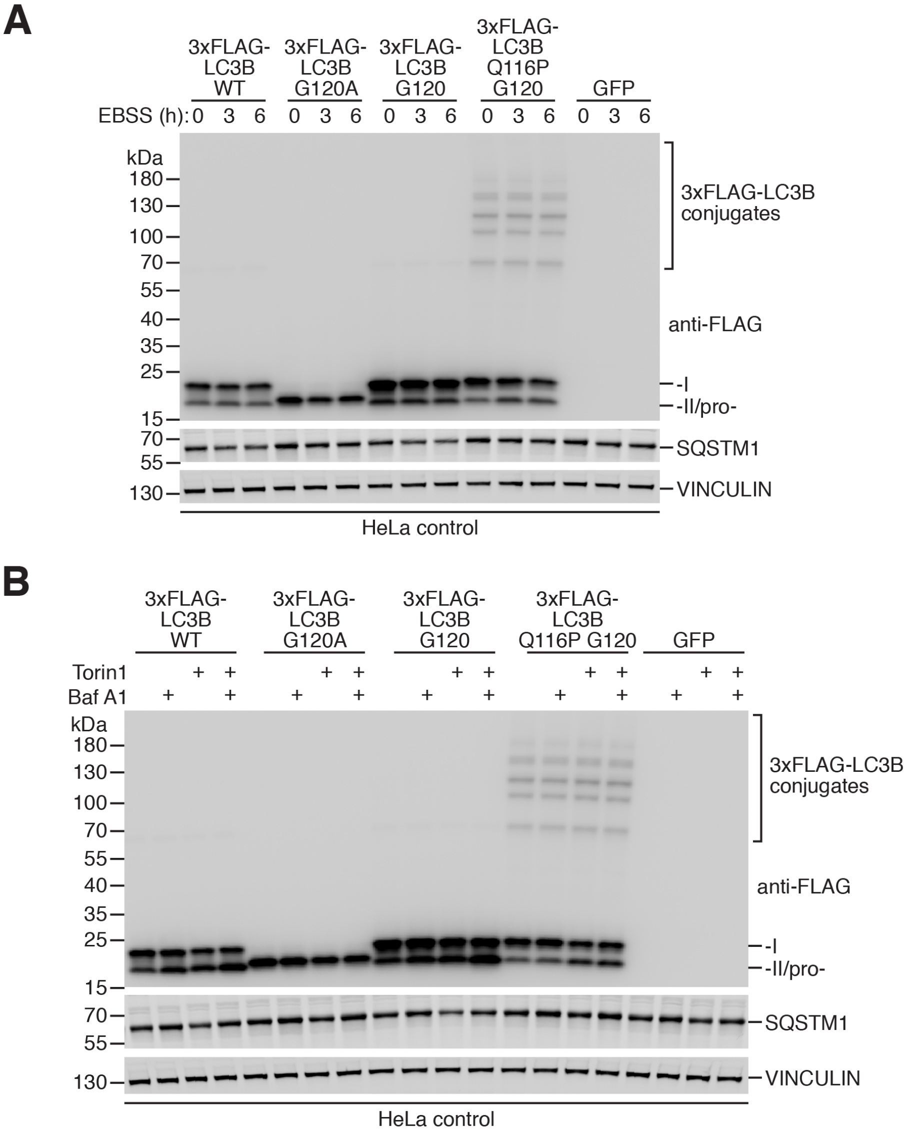

Figure S1. 3xFLAG-LC3B conjugate formation is unaffected by autophagy modulation.

(A) HeLa control cells were transfected with 3xFLAG-LC3B constructs or GFP as a transfection control, prior to washing and incubating in EBSS for 0, 3 or 6 hours to induce amino acid and serum starvation. Lysates were subject to western blotting using anti-FLAG antibody, SQSTM1 as an autophagy marker and VINCULIN as a loading control.

(B) HeLa control cells transfected with indicated constructs were subjected to combinations of pharmacological treatments to induce (Torin1, 250 nM) and/or inhibit (baf A1, 10 nM) autophagy for 3 hours prior to harvesting, with equivalent concentrations of DMSO used in untreated or single-treated samples. Lysates were analyzed by western blotting as for (A).

Figure S2

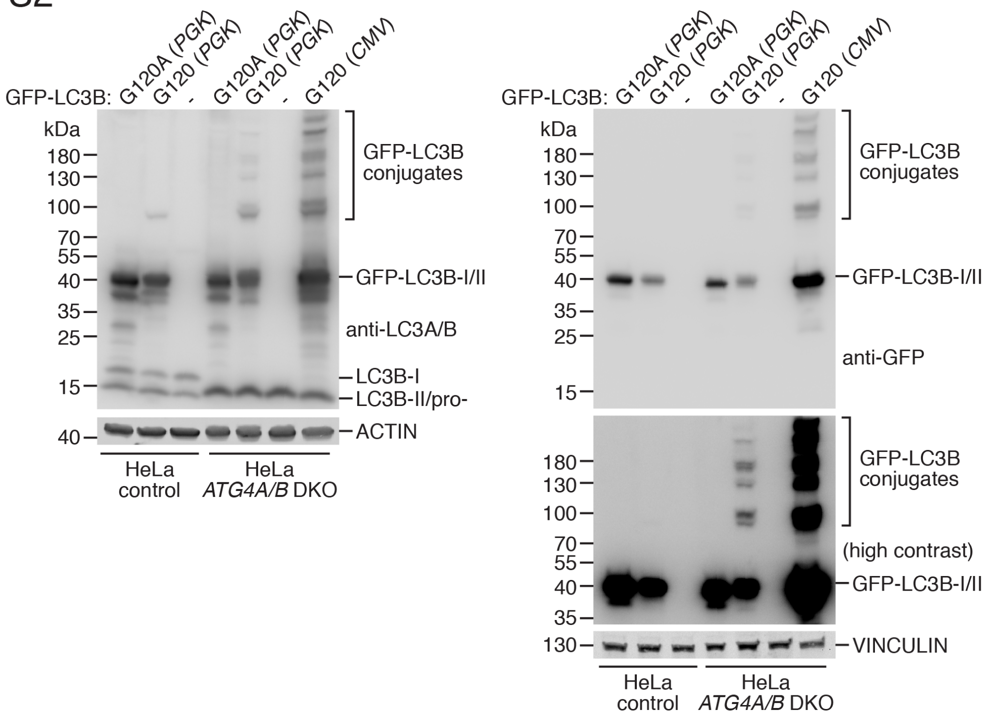

Figure S2. GFP-LC3B G120 forms conjugates in ATG4-deficient cells even when expressed at levels comparable to endogenous LC3B.

Western blotting of lysates from HeLa control and *ATG4A/B* DKO cells (- = untransduced) stably expressing *PGK* promoter-driven GFP-LC3B G120 or GFP-LC3B G120A. HeLa *ATG4A/B* DKO cells stably expressing *CMV*-driven GFP-LC3B G120 were used as a positive control. GFP-LC3B and conjugates were detected using anti-GFP antibody. Anti-LC3A/B antibody was used to detect both GFP-LC3B and endogenous LC3B in the same samples run on a separate gel. ACTIN and VINCULIN were used as loading controls.

Figure S3

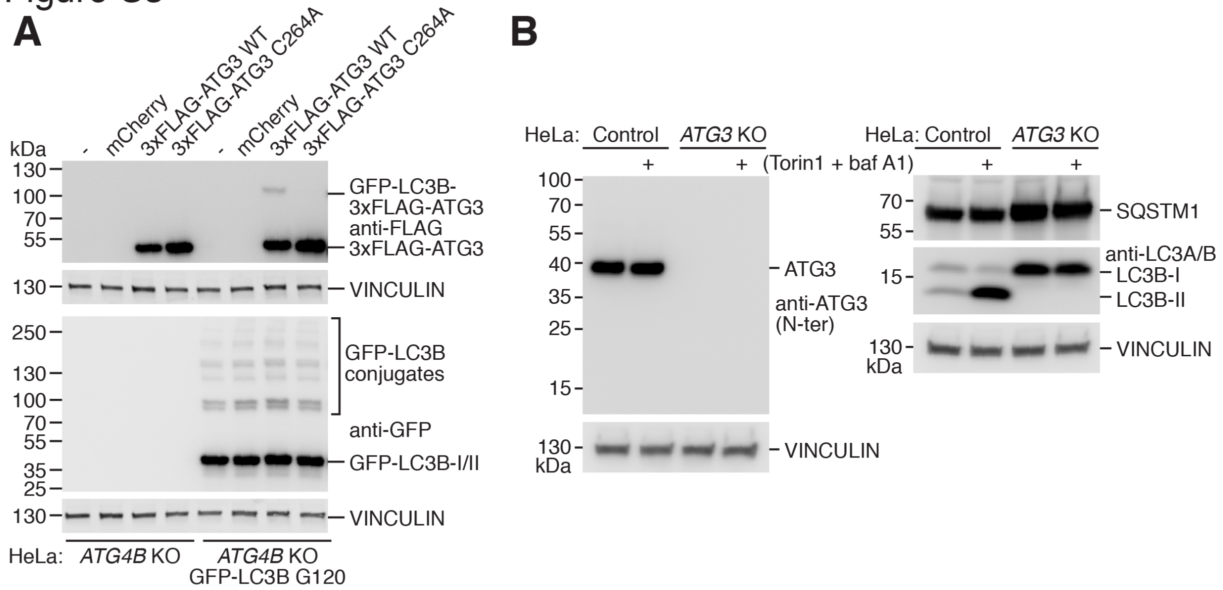

C Human ATG3 protein sequence:

MQNVINTV**K**G**K**ALEVAEYLTPVL**K**ES**K**F**K**ETGVITPEEFVAAGDHLVHHCPTWQWATGEEL**K**V**K**AYLPTG**K**QFLVT**K**NVP
CY**K**RC**K**QMEYSDELEAIIEEDDGDGGWVDTYHNTGITGITEAV**K**EITLEN**K**DNIRLQDCSALCEEEEDEDEGEAADMEEY
EESGLLETDEATLDTR**K**IVEAC**K**A**K**TDAGGEDAILQTRTYDLYITYD**K**YYQTPRLWLFGYDEQRQPLTVEHMYEDISQDH
V**KK**TVTIENHPHLPPPPMCSVHPCRHAEVM**KK**IIETVAEGGGELGVHMYLLIFL**K**FVQAVIPTIEYDYTRHFTM*

ATG3 1-13KR:

K9R	K62R	K86R
K11R	K64R	K124R
K24R	K71R	K131R
K27R	K77R	
K29R	K83R	

ATG3 14-22KR:

K177R	K243R
K183R	K271R
K185R	K272R
K208R	K295R
K242R	

ATG3 allKR:

K9R	K62R	K86R	K177R	K243R
K11R	K64R	K124R	K183R	K271R
K24R	K71R	K131R	K185R	K272R
K27R	K77R		K208R	K295R
K29R	K83R		K242R	

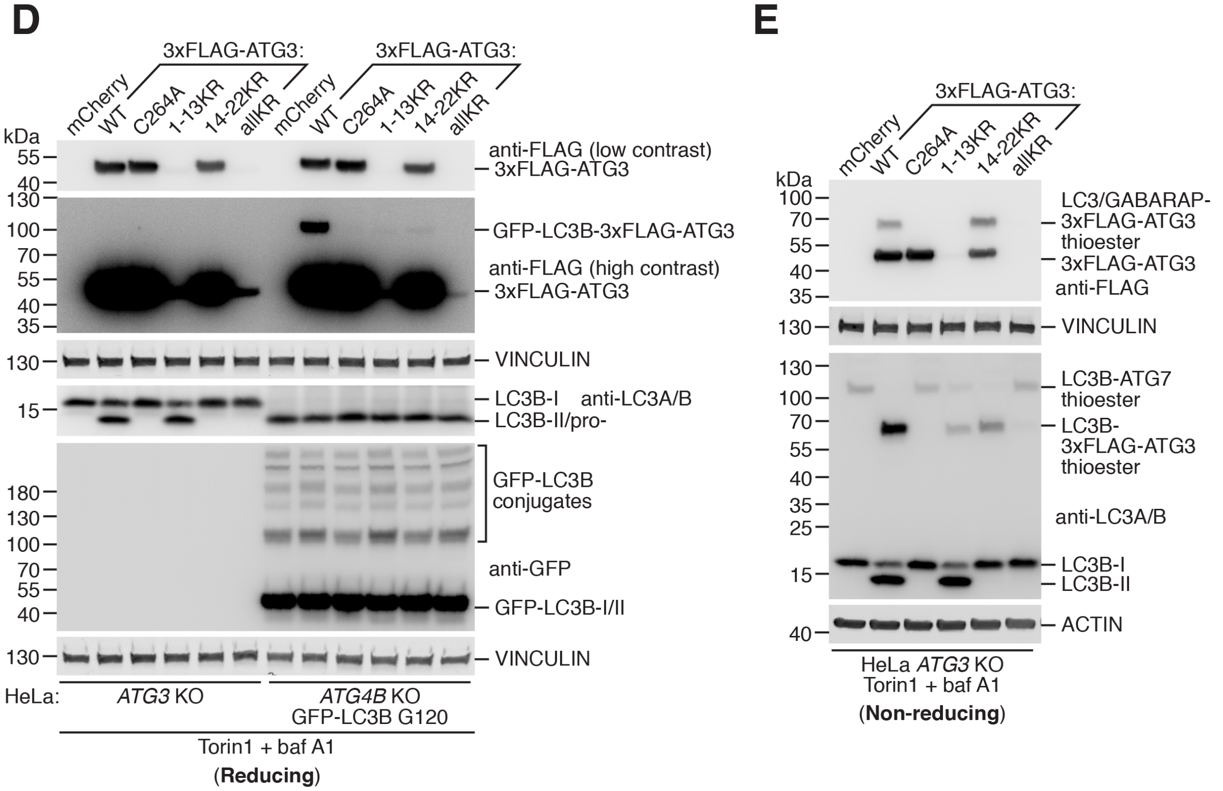

Figure S3. Validation and use of cellular reconstitution systems to determine sets of ATG3 lysine residues required for LC3B-ATG3 conjugate formation, LC3B-ATG3 thioester formation and LC3B lipidation.

(A) Specific reconstitution of the non-thioester conjugate between GFP-LC3B-G120 and transiently expressed 3xFLAG-ATG3 WT. HeLa *ATG4B* KO and *ATG4B* KO cells stably expressing GFP-LC3B G120 were transfected with 3xFLAG-ATG3 constructs, prior to western blot analysis under reducing conditions to disrupt thioester-linked species.

(B) Western blot validation of HeLa *ATG3* KO cells. HeLa control and *ATG3* KO cells were treated with DMSO or 250 nM Torin1 and 10 nM baf A1 for 3 hours prior to harvesting and western blot analysis to assess endogenous LC3B lipidation, SQSTM1 protein level and ATG3 protein level.

(C) Key for mutations present in 3xFLAG-ATG3 constructs used in (D) and (E). Wild-type human ATG3 protein sequence is shown, with the 22 lysine (K) residues in bold. The first 13 lysines are shown in green and the last 9 are shown in magenta. The specific residue positions of lysine to arginine mutations are given in the box below each mutation set name.

(D) Effect of mutating lysine residue sets in 3xFLAG-ATG3 on: rescuing LC3B lipidation in *ATG3* KO cells, and reconstituting GFP-LC3B-3xFLAG-ATG3 conjugate formation in *ATG4B* KO cells stably expressing GFP-LC3B G120. Cells were transfected with indicated constructs and treated for 3 hours with 250 nM Torin1 and 10 nM baf A1 prior to lysis in lysis buffer lacking NEM. Western blotting was performed under reducing conditions to disrupt thioester-linked species.

(E) Effect of mutating lysine residue sets in 3xFLAG-ATG3 on its ability to form a thioester linkage with endogenous LC3/GABARAP. HeLa *ATG3* KO cells were transfected with indicated constructs and treated for 3 hours with 250 nM Torin1 and 10 nM baf A1 prior to lysis in lysis buffer lacking NEM. Western blotting was performed under non-reducing conditions to preserve thioester-linked species.
